# An Assessment of Serum Selenium Concentration in Women with Ovarian Cancer

**DOI:** 10.3390/nu15040850

**Published:** 2023-02-07

**Authors:** Marek Kluza, Sylwia Paszek, Katarzyna Kluza, Sławomir Januszek, Natalia Potocka, Marzena Skrzypa, Alina Zuchowska, Andrzej Wróbel, Piotr Baszuk, Wojciech Marciniak, Marcin Misiek, Jan Lubiński, Jacek Gronwald, Izabela Zawlik, Tomasz Kluz

**Affiliations:** 1 Department of Gynecology, Gynecology Oncology and Obstetrics, Fryderyk Chopin University Hospital, 35-055 Rzeszow, Poland; 2 Laboratory of Molecular Biology, Centre for Innovative Research in Medical and Natural Sciences, Medical College of Rzeszow University, 35-959 Rzeszow, Poland; 3 Second Department of Gynecology, Medical University of Lublin, 20-090 Lublin, Poland; 4 International Hereditary Cancer Center, Department of Genetics and Pathology, Pomeranian Medical University in Szczecin, ul. Unii Lubelskiej 1, 71-252 Szczecin, Poland; 5 Read-Gene S.A., ul. Alabastrowa 8, 72-003 Grzepnica, Poland; 6 Department of Gynecology, Holy Cross Cancer Center, 25-734 Kielce, Poland; 7 Institute of Medical Sciences, Medical College of Rzeszow University, 35-959 Rzeszow, Poland

**Keywords:** ovarian cancer, selenium, serum selenium level, micronutrients, ovarian cancer

## Abstract

Background: Available studies on the effect of serum selenium levels on the risk of malignancies show some conflicting results. In this study, we investigated the correlation between serum selenium levels and ovarian cancer occurrence. Methods: 314 women (157 diseased patients and 157 healthy ones) matched in terms of age and BMI were included in the study. The measurements of selenium in the collected blood samples were performed using an ICP mass spectrometer. Univariable and multivariable analyzes were performed to determine the relationship between the factors under the study and the occurrence of ovarian cancer. Results: The mean concentration of selenium was lower among diseased ones than among controls (53.31 μg/L vs. 78.99 μg/L). A decrease in selenium concentration was noticed with the advancement of ovarian cancer. In univariable and multivariable analyzes, a clear relationship between low selenium concentration and the occurrence of ovarian cancer was found (35.3 (95% CI: 11.2–111; *p* < 0.001) and 45.8 (95% CI: 12.8–164; *p* < 0.001)). Conclusion: The studied patients with ovarian cancer are characterized by statistically significant lower serum selenium levels than patients from the control group. Among the study group, a decrease in selenium concentration was observed with an increase in the FIGO stage. The determination of the role of selenium as a prophylactic factor in ovarian cancer requires further prospective studies.

## 1. Introduction

Selenium is one of the micronutrients of particular significance for the functioning of the human body. It takes part in the regulation of many physiological processes: antioxidant processes, neurotransmitters, immunological processes, thyroid function and reproductive processes [1,2,3]. In 1973, Flohe et al. discovered that selenium is a part of the active center of glutathione peroxidase, which has a great meaning in the antioxidant system [4]. Since then, about 25 selenoproteins, which contain at least one selenocysteine, have been described. Some expression alterations of selenoproteins have been associated with the risk of cancer development and progression in humans (selenoprotein P, glutathione peroxidase and Sep15) [5,6,7,8]. The activity of selenoproteins is optimal with the appropriate plasma level of selenium. The WHO recommends selenium consumption for adults in the amount of at least 55 µg/day. At the same time, it should be noted that selenium is characterized by a narrow range of therapeutic concentrations and that adverse health effects occur both in the states of deficiency and excess of this element. In the literature, these relationships are presented as a U-shaped diagram of selenium activity [1]. Poland is one of the regions with low selenium consumption. This is associated with the risk of a widespread deficiency of this micronutrient, which, as mentioned above, leads to reduced selenoprotein activity and a possible increased risk of developing some malignant neoplasms. In recent years, selenium, like other micronutrients (copper, zinc, iron, cadmium), has aroused increasing interest due to its potential role in carcinogenesis [9,10,11,12,13,14,15,16,17,18]. There is well-documented evidence of a variety of selenium anticancer activity, related both to the function of selenoproteins and the action of selenium and its metabolites in the human body. Most potent chemopreventive effects have been attributed to compounds where the Se moiety is methylated, especially CH3SeH. The available research results indicate the influence of selenium and its compounds on DNA damage repair processes, DNA methylation, an increase in cell apoptosis, an inhibition of cell proliferation and angiogenesis, and by regulating the activity of selenoproteins, selenium affects the reduction in oxidative stress and inflammatory processes and induces phase II conjugating enzymes, supporting the neutralization of carcinogens and reducing the formation of DNA adducts [19,20,21,22,23,24,25,26,27,28,29,30,31,32,33]. It should be noted that the increase in selenoprotein expression in response to increased selenium intake depends on an individual ability [34]. This phenomenon can be at least partly explained by nucleotide polymorphism in selenoprotein genes [35,36,37]. In addition, some of the selenoproteins (TR1, Sep15 and GPx2), apart from anticancer activity, may also contribute to cancer [38,39,40].

Reports on the influence of selenium levels on the incidence of, among others, breast cancer, prostate cancer, lung cancer, liver cancer, stomach cancer, colorectal cancer, cervical cancer and endometrial cancer are available [15,41,42,43,44,45,46,47,48,49,50,51].

Ovarian cancer, as the third most common cancer of the female reproductive system, is also burdened with the highest mortality. In recent years, a slight decrease in the number of cases has been observed. In 2020, there were approximately 313,959 new cases worldwide and about 207,252 deaths due to ovarian cancer [52]. In Poland, these figures in 2019 were 3710 and 2777, respectively [53]. Europe is one of the regions with the highest ovarian cancer incidence in the world.

The already known risk factors for ovarian cancer include, among others, genetic predisposition, age, childlessness, late menopause, early menarche and infertility treatment. Pregnancy, breastfeeding, oral contraception and early menopause are perceived as factors reducing the risk of developing ovarian cancer [54,55,56,57,58]. The protective effect of oral contraception does not translate into a similar effect of menopausal hormone therapy, for which two large studies (The Women’s Health Initiative and the Million Women study) indicate no positive effect or even a possibility of increasing the risk of disease development [59,60,61]. Due to its biology and the lack of specific early symptoms, the diagnosis of ovarian cancer is, in most cases, made at advanced stages of the disease. About 60–70% of cases are diagnosed in FIGO III and IV stages [62,63]. Late diagnosis translates into a worse prognosis for patients. The five-year survival rate for patients with stage I and II disease is 80–90%, dropping to 30–50% for patients with stage III and IV. Despite progress in knowledge about the biology of this cancer, markers of high specificity and sensitivity for ovarian cancer have not been introduced into medical practice. In spite of its limitations, plasma Ca125 levels are the most commonly used biomarkers of ovarian cancer. They are not applicable as a screening test (87% specificity, 70% sensitivity) [64]. Moreover, there was no evidence of the effect of ultrasound screening examinations of the female reproductive organ on the advancement of the disease at the time of diagnosis [65,66,67]. The limitations of available screening methods contribute to great interest in the possible identification of new biological compounds in ovarian cancer diagnostics and prevention.

The role of microelements in ovarian cancer oncogenesis has been the subject of intense research. Apart from selenium, the greatest attention is paid to copper, zinc and manganese [68,69,70,71,72]. The results of the available studies remain inconclusive. The aim of our study is to evaluate the relationship between the level of selenium in women and the probability of ovarian cancer, taking into account the stage of the cancer.

## 2. Materials and Methods

A total of 314 women living in south-eastern Poland were included in the study. The study group consisted of 157 patients with newly diagnosed ovarian cancer treated in the Department of Gynecology, Oncological Gynecology and Clinical Obstetrics, Provincial Hospital No. 1 in Rzeszow during 2015–2019. All diagnoses of ovarian cancer were confirmed using intraoperative histopathological examination. The control group consisted of 157 patients of the clinic, who were admitted to the hospital for diagnostic curettage of the cervical canal and uterine cavity. The diagnoses among the control group were urinary incontinence and uterine fibroids. At the time of admission to the clinic, all patients from the control group were subject to a trans-vaginal ultrasound examination, which excluded any appendages, tumors or presence of ascites. The additional condition for inclusion in the control group was obtaining a negative oncological result of the histopathological examination of the material from curettage. Control group patients were matched with the study group in terms of age and BMI. Before enrollment for the study, all patients signed written consent to participate in the research. The study received the approval of the Bioethics Committee of the Regional Medical Chamber in Rzeszow. The participants completed a questionnaire on age, weight, symptoms, illnesses and operations, medications used, stimulants and family history of cancer. The study was conducted in accordance with the Helsinki Declaration.

### 2.1. Sample Collection and Storage

Venous blood samples for selenium determination were taken at the time of obtaining the results of the intraoperative histopathological examination. The blood was collected in Serum Z/7.5 mL tubes. Prior to centrifugation at 1300× *g* for 5 min, the collected samples were incubated between 30 and 120 min at room temperature. The serum was collected into a new tube and centrifuged again under the same conditions, and finally, it was moved into cryovials and stored in the freezer at −80 °C until the next procedure. The samples from the control group were collected during hospitalization at the clinic and were subject to the same procedures as the samples from patients with ovarian cancer.

### 2.2. Measurement Methodology

ICP mass spectrometer ELAN DRC-e (PerkinElmer, Waltham, MA, USA) was used in order to determine selenium (^80^Se). The instrument was fine-tuned to meet the manufacturer’s requirements prior to the start of each analytical run. Oxygen was selected as a reaction gas. Technical details are available on request. The spectrometer was calibrated using an external calibration technique. A total of 10 µg/mL Multi-element Calibration Standard 3 (PerkinElmer, Waltham, MA, USA) was dilated with blank reagent to predetermined concentrations (1; 2; 5; 10; 50 µg/L) in order to prepare fresh calibration standards daily. Correlation coefficients for calibration curves were always greater than 0.999. To achieve maximal reduction of matrix effects on the Se signal, two techniques were used: the matrix-matched calibration technique and the internal standard technique. Rhodium ^105^Rh was selected as the most appropriate internal standard. According to the analysis protocol, the serum was diluted 30 times in blank reagent. The blank reagent consisted of high-purity water (>18 MΩ), TMAH (AlfaAesar, Haverhill, MA, USA), Triton X-100 (PerkinElmer), n-butanol (Merck, Darmstadt, Germany) and EDTA (Sigma Aldrich, St. Louis, MI, USA).

### 2.3. Quality Control

A certified reference material (CRM), Clincheck Plasmonorm Serum Trace Elements Level 1 (Recipe, German) was used to evaluate the accuracy and precision of measurements. Recovery rates for ^80^Se were between 96 and 110%, and calculated precision (Cv %) was 6.04%. The LOQ method was calculated to be 0.113 µg/L. Additionally, all patients filled out the questionnaire on health status and lifestyle.

### 2.4. Statistics

Patients diagnosed with ovarian cancer and patients of clinics without pathology of the appendages were assigned to one of two equal groups determined on the basis of the distribution of selenium concentrations in the population included in the study. The ranges of selenium concentrations determined in this way were used to analyze the entire population and separate subgroups (FIGO I–II and FIGO III–IV). The patients who qualified for the study were analyzed in terms of selenium concentration (divided into halves with increasing concentration), menopausal status (yes/no), number of deliveries (0/1–2/3 and more), breastfeeding (yes/no), smoking (yes/no), diabetes (yes/no) and hypothyroidism (yes/no). The objective of the analysis was an assessment of the relationship between the abovementioned parameters and the occurrence of ovarian cancer, with particular emphasis on the effect of selenium concentration among the women under the study. The characteristics of the study population are detailed in Table 1. Due to the low number of subjects, factors such as hyperthyroidism, contraception, hormone replacement therapy and endometriosis were excluded from the statistical analysis. The selected division into subgroups (FIGO I–II and FIGO III–IV) was an attempt to identify patients with disease limited to the pelvis minor, usually in good general condition (FIGO I and II), general disease within the abdominal cavity or the whole organism and in worse condition, with often occurring deficiencies in the proper nutrition of the body (FIGO III and IV).

In order to estimate the odds ratio (OR), confidence intervals (95% CI) and determine the test probability (p), uni- and multivariable conditional logistic regression models were applied. All calculations were performed using the R statistical environment (R 4.0.4).

## 3. Results

The mean selenium concentration for all patients (*n* = 314) was 69.12 µg/L. In the analysis concerning the entire population under the study, the average selenium concentration in the half with the lowest selenium concentrations (range 26.84–65.59 μg/L) was 50.11 μg/L, while in the half with the highest selenium concentrations (range 65.67–170.76 μg/L), it was 82.20 μg/L. In the population under the study, both in univariable and multivariable analysis, an increased incidence of ovarian cancer was noted among patients with the lowest selenium levels (range 26.8–65.59)—OR: 35.3 (95% CI: 11.2–111; *p* < 0.001) and 45.8 (95% CI: 12.8, 164; *p* < 0.001). The reference half included 18 patients diagnosed with FIGO stage I or II ovarian cancer and 9 with FIGO stage III or IV cancer. The results of the analysis for the entire population are presented in Table 2.

In relation to the remaining factors, the results of the analyzes indicate a lower risk of ovarian cancer in patients with a history of more deliveries. The odds ratio of ovarian cancer among patients with a history of one–two deliveries was 0.26 (95% CI: 0.10–0.70; *p* = 0.006) in the univariable analysis and 0.09 (95% CI: 0.01; 0.64; *p* = 0.016) in the multivariable analysis. For the group with a history of at least three deliveries, the obtained values were OR 0.24 (95% CI: 0.08–0.66; *p* = 0.006) and OR 0.05 (95% CI: 0.00–0.46; *p* = 0.009), respectively. For the remaining factors, the results were not statistically significant.

**Figure 1:**
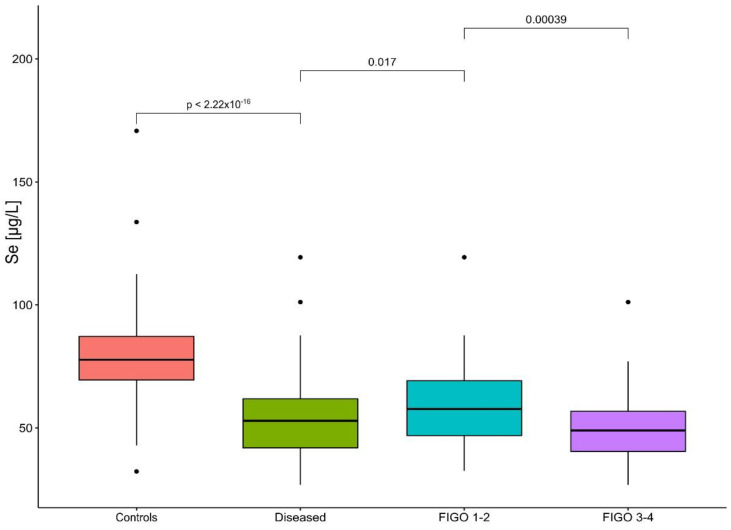
Distribution of selenium level for study subjects. Dots visible on the figure present outliers.

The distribution of selenium concentrations and their median for the control group and the diseased group, divided into separate subgroups, is presented in Figure 1.

The mean and median concentration for the control group were 78.99 and 77.72 μg/L, respectively, and for the women diagnosed with ovarian cancer, 53.31 and 52.89 μg/L, respectively. Among the cases, the mean selenium level decreased with the advancement of the neoplastic process. The mean concentration and median for the stages of FIGO I–II were 59.20 and 57.86 μg/L, and for the FIGO III and IV subgroup, 49.86 and 48.86 μg/L, respectively. In the study subgroups, univariable and multivariable analyzes show an increased incidence of ovarian cancer in patients with a lower selenium concentration. The odds ratio for the FIGO I–II group and the matched controls was 33.0 for the univariable analysis (95% CI, 4.51–241; *p* < 0.001) and 36 (95% CI, 4.38–296; *p* < 0.001) for the multivariable analysis. For the FIGO subgroup III and IV and the matched controls, the OR was 36.5 (95% CI 8.96–149, *p* < 0.001) and 86.6 (95% CI, 4.38–296; *p* < 0.001), respectively. The results of the analyzes in the subgroups are presented in Table 3 and Table 4.

## 4. Discussion

The results of the study show that there is a relationship between the concentration of selenium and the occurrence of ovarian cancer. Both for the entire study group and for the selected subgroups, we showed significant differences between the concentration of selenium among control and case patients. Patients suffering from ovarian cancer were characterized by significantly lower levels of selenium compared to the control group. This relationship also applied to the patients with cancer diagnosed at an early stage. The inverse dependence between the advancement of ovarian cancer and the concentration of selenium seemed to be consistent with disease progression and the associated deteriorating general condition. The study population belongs to the group with a low initial selenium level. More recent data indicate the range of 70–90 µg/L as the optimal selenium concentration among Polish women [73]. The mean selenium concentration in our study for the diseased patients was 53.31 µg/L and 78.99 µg/L for the controls. In the research by Sundstrom, the average concentration of selenium for patients from the study group was 73.2 µg/L [74]. In the research by Caglayan, it was 95.36 µg/L [72], while the average concentration of selenium in the SELECT and Nutritional Prevention of Cancer Trial was approximately 135 [47] and 114 µg/L [75], respectively. Our paper is limited by the relatively small number of patients diagnosed with FIGO I and II stages, but this problem results from the biology of the ovarian cancer itself and diagnostic difficulties in the early stages of this disease.

The potential impact of differences in serum selenium concentration as a risk factor for cancer development has been intensively examined in recent years. We do not know the full role of selenium and selenoproteins in the process of oncogenesis, and the presented research results may be contradictory. The research findings on selenium and ovarian cancer remain unclear. In the 1980s, Sundstrom et al. analyzed the concentration of selenium in patients suffering from gynecological neoplasms in a series of works [74,76,77,78]. They showed that women with diagnosed gynecological cancer had lower mean selenium concentrations than in the control group. In one of the studies, 40 patients diagnosed with ovarian cancer were recruited [74]. The patients with ovarian cancer had significantly (*p* < 0.001) lower selenium concentrations (0.93+/−0.04 mumol/L) than controls matched for age, weight and place of residence (1.22+/−0.03 mumol/L). In stage IV of the disease, the selenium concentration was lower (0.82+/−0.07 mumol/L) than in stages I and II in total (1.00+/−0.04 mumol/L). The concentration of selenium also tended to depend on the effects of treatment, increasing in patients achieving remission of the disease and decreasing in patients with progressive disease (possibly for nutritional reasons). Das analyzed the concentration of selenium in patients with ovarian cancer in the Singapore population [79]. In the conclusions of this study, it was suggested that there is an inverse relationship between selenium concentrations and cases of ovarian cancer. Serum selenium concentrations determined using a modified simple fluorometric method for the control and study groups were 116.7 ± 18.4 and 105.6 ± 15.6 μg/L, respectively. Helzlsouer et al., in their prospective study, showed a relationship between serum selenium concentration and the incidence of ovarian cancer only among patients who were diagnosed at least 4 years after blood sampling (*p* for trend = 0.02) [80]. In 2019, Turkish authors studied micronutrient concentrations in patients with ovarian cancer [72]. They described lower mean selenium concentrations for the study group compared to the control group and noted the increased plasma copper/selenium ratio in ovarian cancer patients. Drózdz et al. analyzed selenium concentrations in patients with malignant neoplasm of the reproductive system and their family members living on a shared farm [81]. The lowest values of selenium in serum were recorded in the group of patients, while in family members, lower selenium concentrations were also observed than in a control group of women unrelated to the patients. The observations from this study may indicate other differences in selenium concentrations, apart from nutritional reasons, especially among people living on a shared farm. Chinese authors investigated the concentration of selenium in serum and ovarian tissue in patients with benign ovarian lesions and ovarian cancer and in healthy patients [82]. The lowest serum concentrations were observed in the group of patients with malignant ovarian cancer. The highest selenium content was found in the tissue of malignant ovarian lesions. In the conclusions of their work, the authors suggest a relationship between the concentration of selenium in the plasma of patients with ovarian cancer and the protective migration of selenium to the tumor tissue. Different results were presented by Canaz et al., who, in their work, did not show any differences between the selenium content in healthy ovarian tissue, borderline tumors and ovarian cancer [83].

In addition to the analysis of selenium concentrations in the serum, there are also studies on the impact of general selenium consumption on the risk of developing ovarian cancer. Terry et al. published a paper whose results indicated a possible protective effect of a higher dietary selenium intake on cancer risk among women in the African American population and in 11 areas of the United States of America [84]. Women with the highest selenium intakes (>20 μg/d) had an approximately 30% reduction in ovarian cancer risk compared with those who did not take supplementation (OR: 0.67; 95% CI: 0.46–0.97; *p*-trend 0.035). This relationship was more pronounced in active smokers (OR: 0.13; 95% CI: 0.04–0.46; *p*-trend = 0.001). There was no correlation between the amount of selenium consumed with food. In his work, Terry did not determine the initial level of selenium, and the intake of micronutrients was assessed using appropriate questionnaires. Analogous outcomes were presented by Gifkins et al., who conducted a study in New Jersey [69]. The results showed an inverse relationship between the consumption of selenium in food and the risk of ovarian cancer. This study did not show a similar relationship for TAC or other antioxidants. Thomson et al. investigated the relationship between dietary intake and antioxidant supplementation (vitamins C, E, A, selenium, carotenoids) and ovarian cancer among 133,614 postmenopausal women participating in the Women’s Health Initiative study [70]. The consumption of individual components was assessed by means of questionnaires. This prospective study results suggest that there is no correlation between the intake of these elements and a reduction in the risk of ovarian cancer. Pan also failed to show a relationship between selenium supplementation and ovarian cancer risk [38]. The conflicting results of studies on the influence of selenium supplementation on the risk of ovarian cancer development seem to be consistent with the scientific works on the influence of selenium consumption on the risk of other cancers. The Women’s Health Initiative study, which enrolled 145,033 postmenopausal women aged 50–79 years, found no association between selenium consumption and cases of breast cancer. The mean follow-up was 15.5 years [85]. Another important study is the randomized SELECT study. Over 35 thousand men were divided into four groups in terms of the concentration of selenium and vitamin E. There was no association between selenium supplementation and prostate cancer cases, and vitamin E supplementation was related with an increased risk of developing the disease [47]. The Nutritional Prevention of Cancer Trial in the United States by Clark and colleagues investigated the effect of selenium supplementation (200 μg/day) on reducing the risk of skin cancer. In total, 1312 patients were recruited for this study. Selenium supplementation was shown to be associated with a statistically significant increase in the incidence of skin squamous cell carcinoma and the total number of nonmelanoma skin cancer cases. However, a secondary endpoint of the analysis indicated that selenium supplementation resulted in a reduction in overall mortality from epithelial carcinomas/malignant neoplasms (RR −0.5), the total number of cases (RRv0.63) and a reduction in the number of cases of lung cancer (RR −0.54), colorectal cancer (RR −0.54) (RR −0.42) and prostate cancer (RR −0.37). The effect of supplementation depended on the initial selenium concentration and was most pronounced for patients with the basal selenium concentration in the two lowest terciles (<121.6) [75]. Chinese authors indicated a reduction in the risk of hepatocellular carcinoma in the selenium supplementation group [45]. Several studies showed a reduction in the incidence of lung, colorectal and prostate cancers associated with supplementation of 200 μg of selenium per day. In addition, a similar effect of supplementation at the same dose on gastric cancer incidence in people with low selenium concentration at the beginning was demonstrated.

The relationship between selenium concentration and ovarian cancer seems to be complex and depends not only on the neoplastic process itself and its advancement or nutritional status but also on the influence of other factors, including genetic ones [86,87,88]. Selenium itself is also beginning to be seen as a possible component of oncological therapy. There are reports on the positive role of selenium supplementation during systemic treatment in reducing side effects [89,90,91]. Sieja et al. investigated the effect of selenium supplementation on the plasma concentration of this micronutrient in patients undergoing ovarian cancer therapy [92,93]. It showed a positive effect of supplementation on the concentration values obtained during the therapy. Selenium supplementation increased the activity of glutathione peroxidase (GSH-Px) and the number of white blood cells during treatment. Sundstrom also suggested a positive effect of selenium supplementation in patients treated for ovarian cancer [78].

## 5. Conclusions

The results obtained indicate the existence of a relationship between serum selenium level and the occurrence of ovarian cancer. The studied patients with ovarian cancer are characterized by statistically significant lower serum selenium levels than patients from the control group. Among the study group, a decrease in selenium concentration was observed with an increase in FIGO stage at the time of diagnosis.

To determine the role of selenium as a prophylactic factor in ovarian cancer and whether patients with a lowered selenium concentration constitute a potential group that requires increased preventive measures, further prospective studies are required taking into account monitoring serum selenium levels and possible selenium supplementation or dietary intake restriction.

## Figures and Tables

**Table 1:** Clinical characteristics of patient affected with ovarian cancer and controls included in the study.

Characteristics	Overall, *n* = 314 ^1^	Controls, *n* = 157 ^1^	Cases, *n* = 157 ^1^	*p*-Value ^2^
Se (μg/L)				<0.001
I (26.8–65.59)	157 (50%)	27 (17%)	130 (83%)	
II (67–171)	157 (50%)	130 (83%)	27 (17%)	
Age	28.00–84.00 (62.44)	30.00–84.00 (62.38)	28.00–83.00 (62.50)	0.9
BMI	16.82–50.00 (27.10)	18.03–45.91 (27.26)	16.82–50.00 (26.94)	0.5
Smoking				0.7
No	291 (93%)	147 (94%)	144 (92%)	
Yes	23 (7.3%)	10 (6.4%)	13 (8.3%)	
Hypothyroidism				0.9
No	272 (87%)	137 (87%)	135 (86%)	
Yes	42 (13%)	20 (13%)	22 (14%)	
Diabetes				0.3
No	270 (86%)	139 (89%)	131 (83%)	
Yes	44 (14%)	18 (11%)	26 (17%)	
Menopause				0.3
No	39 (12%)	23 (15%)	16 (10%)	
Yes	275 (88%)	134 (85%)	141 (90%)	
Number of deliveries				0.023
No deliveries	33 (11%)	9 (5.7%)	24 (15%)	
1–2 deliveries	127 (40%)	67 (43%)	60 (38%)	
3 and more deliveries	154 (49%)	81 (52%)	73 (46%)	
Breastfeeding				0.4
No	61 (19%)	27 (17%)	34 (22%)	
Yes	253 (81%)	130 (83%)	123 (78%)	

^1^*n* %; range (mean) ^2^ Fisher’s exact test; Wilcoxon rank sum test.

**Table 2:** Statistical results for the entire study population.

	Univariable Conditional Logistic Regression			Multivariable Conditional Logistic Regression ^1^		
Characteristics	OR ^2,3^	95% CI ^2,3^	*p*-Value	OR ^2,3^	95% CI ^2,3^	*p*-Value
Se (μg/L)						
I (26.8–65.59)	35.3	11.2, 111	<0.001	45.8	12.8, 164	<0.001
II (67–171)	—	—		—	—	
Smoking						
No	—	—		—	—	
Yes	1.33	0.56, 3.16	0.5	1.37	0.31, 5.99	0.7
Hypothyroidism						
No	—	—		—	—	
Yes	1.11	0.59, 2.10	0.7	1.03	0.36, 2.93	>0.9
Diabetes						
No	—	—		—	—	
Yes	1.57	0.80, 3.07	0.2	1.44	0.47, 4.44	0.5
Menopause						
No	—	—		—	—	
Yes	3.33	0.92, 12.1	0.067	6.15	0.43, 87.0	0.2
Number of deliveries						
No deliveries	—	—		—	—	
1–2 deliveries	0.26	0.10, 0.70	0.008	0.09	0.01, 0.64	0.016
3 and more deliveries	0.24	0.08, 0.66	0.006	0.05	0.00, 0.46	0.009
Breastfeeding						
No	—	—		—	—	
Yes	0.73	0.40, 1.32	0.3	1.82	0.41, 8.02	0.4

^1^ Multivariable conditional logistic regression models are adjusted for smoking (yes/no), hypothyroidism (yes/no), diabetes (yes/no), menopause (yes/no), number of deliveries (no deliveries/1–2 deliveries/3 and more deliveries), breastfeeding (yes/no). ^2^ Fisher’s exact test; Wilcoxon rank sum test; ^3^ OR = odds ratio; CI = confidence interval.

**Table 3:** Statistical results for the FIGO I–II subgroup.

	Univariable Conditional Logistic Regression			Multivariable Conditional Logistic Regression ^1^		
Characteristics	OR ^2,3^	95% CI ^2,3^	*p*-Value	OR^2,3^	95% CI ^2,3^	*p*-Value
Se (μg/L)						
I (26.8–65.59)	33.0	4.51, 241	<0.001	36.0	4.38, 296	<0.001
II (67–171)	—	—		—	—	
Smoking						
No	—	—		—	—	
Yes	3.00	0.61, 14.9	0.2	2.76	0.21, 36.9	0.4
Hypothyroidism						
No	—	—		—	—	
Yes	1.00	0.25, 4.00	>0.9	2.91	0.34, 24.7	0.3
Diabetes						
No	—	—		—	—	
Yes	1.50	0.42, 5.32	0.5	1.68	0.26, 10.6	0.6
Menopause						
No	—	—		—	—	
Yes	2.00	0.18, 22.1	0.6	2.27	0.00, 4.541	0.8
Number of deliveries						
No deliveries	—	—		—	—	
1–2 deliveries	— *	0.00, —	>0.9	0.00	0.00, —	>0.9
3 and more deliveries	— *	0.00, —	>0.9	0.00	0.00, —	>0.9
Breastfeeding						
No	—	—		—	—	
Yes	0.60	0.14, 2.51	0.5	9.57	0.08, 1.106	0.4

^1^ Multivariable conditional logistic regression models are adjusted for smoking (yes/no), hypothyroidism (yes/no), diabetes (yes/no), menopause (yes/no), number of deliveries (no deliveries/1–2 deliveries/3 and more deliveries), breastfeeding (yes/no). ^2^ Fisher’s exact test; Wilcoxon rank sum test; ^3^ OR = odds ratio; CI = confidence interval; * non-calculable due to low number of subjects.

**Table 4:** Statistical results for the FIGO III–IV subgroup.

	Univariable Conditional Logistic Regression			Multivariable Conditional Logistic Regression ^1^		
Characteristics	OR ^2,3^	95% CI ^2,3^	*p*-Value	OR^2,3^	95% CI ^2,3^	*p*-Value
Se (μg/L)						
I (26.8–65.59)	36.5	8.96, 149	<0.001	86.6	13.6, 554	<0.001
II (67–171)	—	—		—	—	
Smoking						
No	—	—		—	—	
Yes	0.86	0.29, 2.55	0.8	0.43	0.03, 5.28	0.5
Hypothyroidism						
No	—	—		—	—	
Yes	1.14	0.56, 2.34	0.7	0.45	0.09, 2.22	0.3
Diabetes						
No	—	—		—	—	
Yes	1.60	0.73, 3.53	0.2	2.04	0.40, 10.3	0.4
Menopause						
No	—	—		—	—	
Yes	4.00	0.85, 18.8	0.080	20.1	0.04, 10,933	0.4
Number of deliveries						
No deliveries	—	—		—	—	
1–2 deliveries	0.38	0.13, 1.07	0.066	0.20	0.02, 1.89	0.2
3 and more deliveries	0.27	0.09, 0.80	0.019	0.03	0.00, 0.68	0.027
Breastfeeding						
No	—	—		—	—	
Yes	0.76	0.40, 1.46	0.4	1.34	0.24, 7.40	0.7

^1^ Multivariable conditional logistic regression models are adjusted for smoking (yes/no), hypothyroidism (yes/no), diabetes (yes/no), menopause (yes/no), number of deliveries (no deliveries/1–2 deliveries/3 and more deliveries), breastfeeding (yes/no). ^2^ Fisher’s exact test; Wilcoxon rank sum test; ^3^ OR = odds ratio, CI = confidence interval.

## Data Availability

The data presented in this study are available on request from the corresponding authors.
